# A mobile game to promote ART adherence among adolescents living with HIV in Eswatini: Development and prototype testing of “The Conqueror”

**DOI:** 10.1371/journal.pone.0321907

**Published:** 2026-02-05

**Authors:** Londiwe D. Hlophe, Peter S. Nyasulu, Constance S. Shumba

**Affiliations:** 1 Division of Epidemiology and Biostatistics, Faculty of Medicine and Health Sciences, Stellenbosch University, Cape Town, South Africa; 2 Division of Epidemiology and Biostatistics, School of Public Health, Faculty of Health Sciences, University of the Witwatersrand, Johannesburg, South Africa; 3 Division of Epidemiology and Social Sciences, Institute for Health and Equity, Medical College of Wisconsin, Milwaukee, Wisconsin, United States of America; University of Greenwich, UNITED KINGDOM OF GREAT BRITAIN AND NORTHERN IRELAND

## Abstract

**Background:**

In Eswatini, adolescents living with HIV (ALHIV) face disproportionate challenges in achieving optimal anti-retroviral therapy (ART) adherence, as evidenced by a viral load suppression (VLS) of 77.8% among those aged 15–19 years, compared to the 96.2% VLS observed among adults on ART. While mobile phone ownership has surged globally, facilitating the use of mobile phone-based interventions in healthcare, there remains limited evidence on the development and testing of such interventions for improving ART adherence among ALHIV. This study aimed to design a mobile gaming application (app) to promote ART adherence among ALHIV in Eswatini.

**Methods:**

A systematic, iterative approach involving a multidisciplinary team of researchers, ALHIV, and app developers was adopted in designing, developing, and testing the prototype of “*The Conqueror” game*. A total of seven focus group discussion sessions, each comprising 12 participants conveniently sampled from five Teen Clubs in the Hhohho region of Eswatini, were conducted between 27 April and 20 July 2024 to inform the design and development of the mobile game. The usability and acceptability of the game were assessed using self-administered questionnaires among 35 ALHIV, who were not involved in the game development process and were conveniently selected from the Teen Clubs. The qualitative data were analysed thematically, while the quantitative data were analysed descriptively.

**Results:**

Participants desired mobile games aimed at enhancing social support through reminders, acceptance, encouragement, and knowledge. Social support was integrated into a storyline that informed the development of *The Conqueror*, which centres on a hero who conquers opposition and collects an artefact daily to maintain viral suppression. The game features gamification elements such as a life bar, progressive difficulty, daily cycles, three stages with five-day challenges, and power-ups for health boosts and speed increases. The feasibility of *The Conqueror* were rated relatively high, at 86.2% and 82.8%, for usability and acceptability respectively.

**Conclusion:**

Gamified mHealth interventions co-developed with potential end-users demonstrate high usability and acceptability. However, further piloting of *The Conqueror* game through randomized trials is essential to evaluate the feasibility and effectiveness of this approach in improving ART adherence among adolescents before broader implementation and scaling to a wider adolescent population.

## Introduction

The introduction of anti-retroviral therapy (ART) has reduced AIDS related deaths by 69% since the peak in 2004 globally [1]. In sub-Saharan Africa (SSA), home to 70% of all the global population living with HIV, there has been a 60% drop in AIDS related death due to the introduction of ART [1,2]. However, adolescents constitute a disproportionately affected population globally, with only 65% of those living with HIV on ART and an estimated 27,000 AIDS-related deaths reported in 2023 [3]. The situation is particularly pronounced in SSA, which is home to approximately 90% of the world’s adolescents living with HIV (ALHIV) [2,4]. For instance, a systematic review of studies conducted between 2010 and 2022, revealed that viral load suppression (VLS) was 55% among ALHIV in SSA [5]. A non-suppressed viral load is associated with opportunistic infections and AIDS related deaths heightening the need for interventions aimed at ART adherence and a suppressed viral load [6–8].

Optimum ART adherence, defined as taking ≥95% of doses consistently and correctly, is a prerequisite for achieving VLS [9]. However, ART adherence is low among ALHIV, particularly those from SSA group highlighting the urgent need for innovative interventions promoting ART adherence among this age group [10]. Various strategies have been implemented to improve adherence among ALHIV, including differentiated models of care such as peer support, enhanced counselling, family-based financial support, adolescent-focused packages of care, and reminders [5]. Despite these efforts, the systematic review cited above found that none of the interventions identified were statistically associated with improved ART adherence among ALHIV in SSA [5].

Nevertheless, the proliferation of mobile phones worldwide, leading to 70% internet connectivity among young people, has facilitated the adoption of mHealth interventions aimed at improving ART adherence [11–15]. Examples of such interventions include text messaging, phone calls, gamification and mobile applications, which have been implemented to support young people living with HIV in SSA [12]. While promising, only two of the six studies conducted among young people in SSA reported statistically significant improvements in ART adherence [12]. Gamified interventions have demonstrated positive outcomes in health promotion, yet few have targeted adolescents despite their high engagement with mobile game [16,17]. For example, of the seven gamified interventions developed for HIV prevention and care, only two were specifically designed for adolescents, and of these, only one incorporated adolescents’ input despite studies emphasizing the importance of user-centred interventions [18]. Furthermore, gamified interventions targeting HIV prevention and care are generally scarce in developing countries, as most research in this area has been conducted predominantly in the United States, neglecting regions that are most affected by the pandemic [18].

In Eswatini, a country with the highest global prevalence of HIV at 24.8%, high VLS of 96% have been reported among the adult population, while only 77.8% among adolescents aged 15–19 years [19,20]. Multiple interventions aimed at improving ART adherence among ALHIV have been implemented. These include the introduction of Teen Clubs, which offer support and ART counselling through peers and lay workers [21]. Peer support is further promoted through peer education and home visits by peers for ALHIV lost to follow-up and those with sub-optimum ART adherence or unsuppressed viral loads [22]. Additionally, reminders such as phone calls by healthcare workers when ALHIV miss a refill date are utilized to encourage ART adherence [21].

The growing trend of mobile phone access suggests a promising opportunity to leverage mHealth interventions to enhance ART adherence among ALHIV in Eswatini. However, the use of mobile health (mHealth) to improve ART adherence among ALHIV has not been extensively explored, despite the country reporting 1.5 million mobile phone subscriptions [23].

To the best our knowledge, no gamified mHealth intervention aimed at promoting ART adherence among ALHIV has been designed in Eswatini. Currently, the only gamified intervention in the country, *SwaziYolo*, focuses on increasing risk perception among young adults aged 18–29 years [24]. Additionally, the *TuneMe Program*, a mobile application, aims to promote positive health behaviours, life skills development, and link young people to health services through motivational content [25]. Moreover, mHealth interventions promoting ART adherence in Eswatini remain limited to short text messaging and phone calls, with little exploration of alternative approaches such as gamified interventions [21]. A study among ALHIV in Eswatini highlighted that adolescents consider gamified interventions feasible, citing widespread mobile access and previous gaming experiences [26]. However, participants emphasized the importance of ensuring confidentiality and accessibility to maximize the intervention’s impact. They further suggested that gamified interventions should focus on providing support, HIV and ART-related education, and knowledge while safeguarding adolescents’ privacy [26].

This study builds on these findings by emphasizing the importance of involving adolescents in the design process. It aims to co-develop a gamified mHealth intervention specifically targeting ART adherence, ensuring that the intervention aligns with the needs and preferences of end-users.

## Materials and methods

### Study design

A mixed-method design was used to develop and test “*The Conqueror”* application. The development of *“The Conqueror”* mobile game prototype followed a co-design process combining user-centred, participatory, and collaborative approaches [27]. This approach guaranteed that the design process was iterative focusing on the needs of ALHIV, and ensuring their active participation together with the researcher and a team of developers [28].

The design of “*The Conqueror”* was underpinned by evidence-based health behaviour change models and the use of persuasive technology to enhance medication adherence. Specifically, the development was guided by the principles of both the Social Cognitive Theory (SCT) and the Information-Motivation-Behavioural Skills (IMB) model of ART adherence [27,29,30]. The SCT was chosen for its comprehensiveness compared to other models and theories of behaviour change [31]. Unlike models like the health belief model that primarily focus on individual factors, SCT considers behaviour as an interplay between the individual and their environment. According to SCT, there is a continuous dynamic interaction among three factors: the individual, the environment, and behaviour [31,32]. In contrast, most other models view behaviour as primarily influenced by individual factors, thereby overlooking critical elements such as social support, self-efficacy, habits, and the interaction of these factors with outcomes [32]. The SCT posits that HIV and ART knowledge serve as gateways for behaviour change, which is further promoted through observational learning and the modelled and vicarious experiences of health behaviours [30,33]. This approach aims to increase self-efficacy, wherein individuals expect certain outcomes and are reinforced (both negatively and positively) for behaviour change [30].

The IMB model, on the other hand, is widely used in ART adherence interventions due to its adaptability for designing targeted adherence strategies [34]. The IMB stipulates that high levels of ART adherence can be achieved when ALHIV possess adequate HIV and ART knowledge, have a high level of personal and social motivation, and receive coaching to help them integrate medication-taking into their daily routines [27,35]. This model underscores the importance of behavioural skills, such as correctly taking medication, managing side effects, and incorporating medication routines into daily life [35]. These skills are essential for translating knowledge and motivation into actual adherence behaviour. While knowledge and motivation are crucial for medication adherence, their true impact is realized through the patient’s ability to effectively manage their ART regimen [27,29]. The IMB model has been successfully applied in previous interventions, particularly mobile health-based initiatives aimed at improving ART adherence among people living with HIV [35–37]. Finally, combining the IMB model with SCT integrates the diverse factors associated with behaviour change that individual theories may overlook. This combination highlights the importance of self-efficacy and behavioural skills, which are critical for sustained ART adherence despite the multifaceted barriers stemming from both intrinsic and extrinsic factors [34].

Persuasive technology, which is the use of applications to influence users’ attitudes, beliefs, and behaviours toward a desired objective (in this case, medication adherence), was also a key component in the design of “*The Conqueror*” [38,39]. The principles of persuasive technology, which focus on enhancing motivation and skills while providing triggers to encourage medication adherence, were incorporated into the game’s design [38]. Persuasive technology has been used successfully in previous ART adherence interventions, yielding positive outcomes [40,41]. To further ensure the effectiveness of “*The Conqueror*” in promoting ART adherence, the game was designed with a strong emphasis on user perspectives. This approach is supported by evidence from successful ART adherence interventions that highlight the importance of involving end users in the development of interventions to ensure their effectiveness and the sustainability of behaviour change, particularly in behaviour change interventions [42–45].

### Settings and participants

The study was conducted among adolescents aged 10–19 years living with HIV and on ART who are members of Teen Clubs in Eswatini. Teen Clubs are a model of differential ART care services found in health facilities which utilized lay workers for the ART adherence counselling, support and medication refill [22,46].

Teen Clubs from five health facilities in the Hhohho region were included. The Hhohho region was purposively selected because of its high club population compared to the other three regions in the country [47]. Teen Clubs were purposively selected with the assistance of the Ministry of Health, Swaziland national AIDS Program (SNAP); *National Paediatrics’ HIV Care and Treatment department* if they had been vibrant for a least two years. Teen Clubs from urban, semi-urban and rural places were selected in this study to ensure representativeness of views of ALHIV from different settings in the country [48].

**Fig 1:**
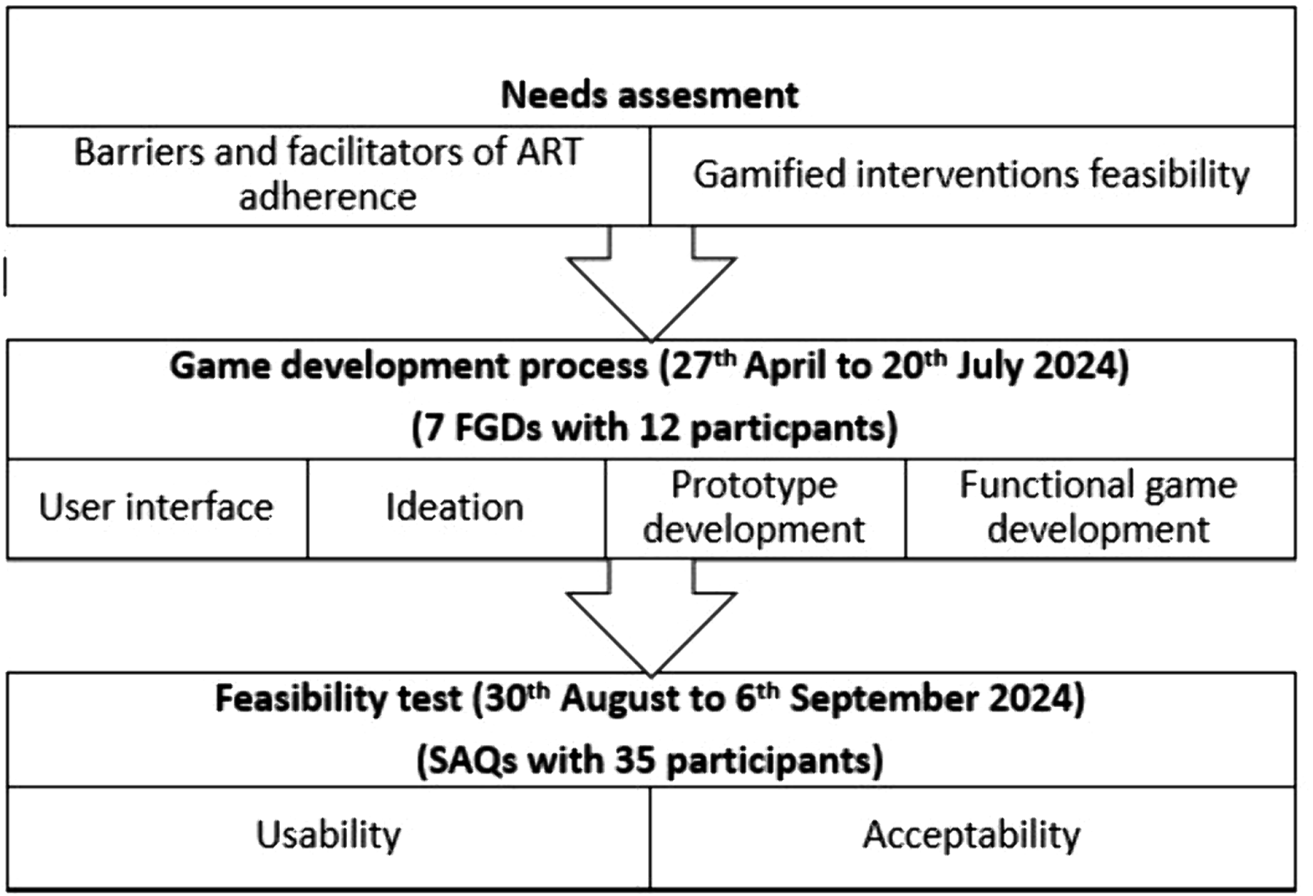
The mobile game development phases.

The game development process involved seven focus group discussion (FGD) sessions, each comprising 12 participants conveniently sampled between March 9 and April 13, 2024. These FGDs were conducted across four phases (phases 1–4) of the game development process (Fig 1). Eligibility criteria included an expressed interest in participating in the game design and development, provision of both guardian and self-consent, availability on all scheduled design dates, and the ability to travel independently to the study site. The sample size was guided by the principle of sample size for FGDs [49]. FGDs took place between April 27 and July 20, 2024.

For usability and acceptability testing (phase 5), 35 participants who were not involved in the game development phases were conveniently sampled based on smartphone ownership and willingness to participate. These participants were recruited between July 6 and August 3, 2024. Usability testing was conducted from August 30 to September 6, 2024.

The game development phases are summarised in Fig 1.

### Ethical considerations

Approval to conduct the study was sought from the Stellenbosch University Health Research Ethics Committee (Reference number: S24/02/037) and Eswatini Health and Human Research Review Board of the Ministry of Health (Protocol Reference Number: EHHRRB143/2023). Also, a letter for permission to conduct the study at each facility was granted by the Ministry of Health, Director of Health Services Office.

Thirdly, consent was sought from participants and caregivers. Participants were informed that participation was voluntary and were free to withdraw from the study should they feel so. Only participants who provided signed consent forms and assents forms were included in the study. The videogame development team signed a non-disclosure agreement form prior to meeting the participants. Participants identified pseudo names they preferred to use during the game development process to ensure their identity was concealed. The pseudo names were placed in front of each participant throughout all the meetings and interviews. For the game usability and acceptability testing, no personal identifiers, including participants’ names or HIV status, were collected to ensure anonymity and prevent unintended disclosure.

### Iterative game development process and results

The development of the game was guided by an extensive literature review and insights from primary and secondary studies conducted by the authors, which highlighted barriers to ART adherence among adolescents [5]. Additionally, a study was undertaken to assess mobile phone ownership, access, and perceptions regarding gamified interventions among ALHIV to confirm the need and relevance of a mobile game intervention [26]. This phase aimed to assess the needs of ALHIV concerning gamified interventions designed to support ART adherence (Fig 1). Findings from this phase informed the game development process.

The game development process involved four distinct phases, during which seven FGDs sessions were held at a central location. Each session lasted approximately 90 minutes and was facilitated by the researcher with the support of an app developer. Participants were provided with transport reimbursements, breakfast, and lunch, and they received a $6 compensation for their time and input. These sessions were instrumental in refining the game concept and ensuring its alignment with the needs and preferences of ALHIV.

Lastly, the developed game prototype was further tested for acceptability and usability. Recruited participants were reimbursed $3 for internet bundles.

## Phase 1: User interface (UI)

### Method

The user interface phase was primarily aimed at identifying strategies that can promote ART adherence among ALHIV. A FGD was conducted guided by a focus group interview guide with a broad open-ended question “what strategies do you think can work to promote ART adherence?” This approach encouraged participants to think critically about potential solutions and share their ideas in an open and collaborative environment. Inclusive prompts were designed to encourage full participation from all participants, including younger age groups, while probing questions were employed to ensure the collection of comprehensive and in-depth data. Data collection was halted when data saturation was reached, when no new information emerged from participants [48].

As the participants discussed various strategies, the researcher actively merged these strategies into meaningful themes in real-time, ensuring that the data collection and thematic analysis were conducted simultaneously [42,50,51]. This iterative process allowed for the identification of key themes and strategies that could be used to inform the design and development of interventions aimed at improving ART adherence among ALHIV. A deductive-inductive data analysis approach was used whereby the initial code frames were guided by the IMB Skills Model and SCT principles, which emphasize the importance of knowledge, behavioural skills, reinforcement, expectations, and self-efficacy in promoting behaviour change [30,36]. Additional themes emerged inductively from the participants’ discussions. Identified themes were presented to participants for approval to enhance confirmability of the findings [49].

## Results

Participants identified social support as the main strategy to promote ART adherence. Social support could be through reminders, acceptance, and de-stigmatization of HIV. Therefore, interventions targeted at social support were reported to have a positive influence on their ART adherence patterns resulting to a suppressed viral load and increased CD4 count as ultimate outcomes. Participants stated that daily taking of medication can be reinforced by positive feedback and rewards from both peers, family members and those close to them. Lastly, information was reported to be critical in ensuring a sustained ART adherence. These are summarised in Table 1 below.

**Table 1:** Strategies to improve ART adherence with their codes and examples by participants.

Theme	Codes	Examples
1. Support	Reminders	“*You know that thing of forgetting is real and it’s even worse when you have no one to remind you and now that I am no longer at home, I have to remind myself and it feels so demotivating at time*” Male ALHIV aged 19
	Acceptance	*“You see things like this make us feel accepted. Like now we are here together from different Clubs, and we getting to know each other and sharing our experiences” Female ALHIV aged 19*
	De-stigmatization of HIV	“*The worst thing is having to hide your status just because you know people will start gossiping about you… If you have a headache, you just say it and people don’t frown at you but the moment you say HIV, it’s like everyone leaves you… people should be taught that HIV is just like a headache*” Female ALHIV aged 19
2. Outcome expectation	Increased CD-4 count and suppressed virus	“*The one thing important is having a high CD-4 and when they tell you that you are now U=U eish the best news ever. laughs*” Female ALHIV aged 16
3. Skill supported	Daily taking of pill	“*The most important thing is taking the pill daily and that sums up to weekly and eventually every day for 365 day a year. If we can do that besides all the challenges, we face we will be fine but it’s hard*” Male ALHIV aged 19
4. Knowledge	HIV information	*“Sometimes you read on the internet of new innovations like now they say there will be an injection you take once a month instead of the daily pill. Problem is it true or not since there is no person to tell us”* Female ALHIV aged 18
	Health knowledge	*“We just need to know more about life in general you know when living with HIV… remember when COVID-19 started, some said we will die because we are already sick and then some said we are safe… it was confusing”* Male ALHIV aged 19
5. Reinforcement	Positive feedback	“*It always feels like they are waiting for you to make a mistake… like can’t they see when I do something right. Missing a pill once a day it’s like I have killed someone when I have been taking them for the past 6 days and nobody notices that*” Female ALHIV aged 15
	Rewards	*“Sometimes you need someone who will praise you or even offer something for a week of complete adherence”* ALHIV aged 19

## Phase 2: Ideation

### Method

The strategies to promote ART adherence identified in Phase 1 were further refined and ideated into a storybook, which served as a foundational narrative for *“The Conqueror”* mobile game.

For the storybook ideation process, participants were divided into two groups, each tasked with developing a storyline that would reflect the strategies identified in Phase 1. Each group was provided with charts and other creative materials to visually demonstrate their storyline. The groups were given one hour to brainstorm, collaborate, and develop their respective storylines. After the breakaway sessions, the groups reconvened to present their storylines to the entire team, including other participants, the researcher, and the software developers. Each group presented their storybook, which was then critiqued by the entire team. This collaborative critique session was aimed at identifying strengths, areas for improvement, and ensuring alignment with the IMB and SCT models of behaviour change.

Following the critique session, the groups were given the opportunity to decide on the most preferred storyline. Through a collaborative decision-making process, the final storyline was selected, which would serve as the narrative basis for *“The Conqueror”* game. The selected storyline was further refined and developed into a comprehensive storybook. This storybook laid the groundwork for the board game prototype, ensuring that the narrative was engaging, relatable, and educational, while also aligning with the principles of behaviour change.

## Results

Two storybooks were presented by the two groups based on their age groups (10–14 years and 15–19 years) based on two themes: daily tasks and the fight associated with living with HIV and being on ART.

a) The daily task of living with HIV and being on ART

The 10–14 years old group presented a storybook based on a life of a young girl who wakes up daily to make a choice of dressing up for the day (taking ART medication) based on the daily challenge (barriers). The daily challenges included going to school, going to church, going to town, and going to a dance competition. To dress up, one is timed (competing activities) to make sure that you meet the challenge. Clothes to be in a closet and you select them based on the challenge and work against time as you dress the child completely (ART adherence). While dressing up, there should be information on how to dress (like eating before taking your medication), the weather (barriers associated with ART taking such as taste, size, and side effects of the pill) and the importance of the event (viral load suppression and high CD4 count). For one to win, they must be able to dress and have a complete facial make-up and hair done based on the daily challenge (maintained suppressed viral). Failure to keep time at each phase of the dressing up, means you cannot access all accessories in the subsequent stage (suppressed viral load) (S1 Fig).

a) The daily fighting when living with HIV

The second group presented a story line on a person who is fighting for a better life each day (ART) while meeting challenges (barriers) along the way. The aim is to stay fit, hydrated, and healthy (suppressed viral load and high CD4 count). Of importance is that along the way will be challenges which at time will cause him/her to be weak (missing daily dozes) but there will be healthy stations for him/her to get assistance (refill and support). There should be encouraging messages and rewards for him/her at every stage of the journey (support) (S2 Fig).

Upon both groups’ presentation, participants coincided that they desired a game where a player is in a journey to keep healthy but along the journey there are different challenges they face (barriers to ART adherence). However, a player needs to overcome and stay healthy (medication adherence). There should be healthy stations along the way to revitalize their health (ART refills). There should be supporting information as player either fails or wins each challenge (words of encouragement and HIV and ART knowledge). Further, players need to collect points along the way which increase their life bar (viral load suppression) and there should be a measure of their life bar as they loss or gain points along the way (VL results and CD4 count). Each level of the game should occur in different settings like real life on an ALHIV with different features. At the finishing point, players should get the total points gained and life bar (pill count and VL result). Therefore, a game which includes a player collecting treasures was agreed upon.

## Phase 3: Prototype development: Questions and answers

### Method

The board game from Phase 2 was developed into an interactive digital wireframe based on the charted storybook. During the prototype development, there were two iterative meetings between the team to ensure that all stated preferences and needs were being incorporated. In both meetings, participants were guided by the game development team through the application installation and given time to explore the application and complete tasks within the application. Participants were asked to think out loud to provide real-time feedback and recommendations. Data collection was stopped when no new suggestions were submitted by participants. The researcher documented participants’ submissions and summarized them thematically, aligning them with the game features identified by the participants. Data analysis was conducted concurrently with data collection. The identified themes were collaboratively reviewed and harmonized by the researcher, participants, and the video game developer to ensure they accurately represented the participants’ feedback and recommendations.

## Results

Based on phase 2 game concept and real-time feedback (Table 2) from participants, “*The Conqueror*” was designed. *The Conqueror* was designed to be played in both mobile (iOs and android) and personal computer (PC) platforms to cater for all adolescents. The Game Maker studio professional engine was used for the design as it allows both PC and mobile platforms, is an open saver allowing for cheaper game download, and can be used for user metrics download [52]. For controls, touch controls for movement are used in mobile phones while the keyboard and mouse controls are used for PC.

**Table 2:** The real-time feedback by participants during the testing of the alpha version of the game.

Category	Feature	Recommendation	Representation	Action
Technical aspect	Visual design element	Visuals changes with scenes or stages	Improved general health as one adheres to medication	Dark and gritty with a gradual shift to brighter, more hopeful visuals as the hero progresses
	Audio design element	Music to change with scenes and should match ambience of the environment in which game is taking place	Improved perspective of life with HIV and being on ART as you conquer daily challenges and adhere to medication.	Eerie, atmospheric music for the dark stages, shifting to more uplifting themes in later stages.
	Privacy or protection feature	There should not be any messages or hint related to medical information	To ensure privacy and prevention of unintended disclosure	Action takes place in environment and not in the body
Gaming mechanics	Progression mechanic	Difficulty index increases with each stage	The challenges as you grow	Increase in speed
	Feedback mechanic	Include messages for every action	Encouragement, support, and acceptance	Messages such as: “*Well done”, “impressive”, “don’t give up”, “You’ve got it”, “the demon is suppressed” “you are amazing”*
	Outcome mechanic	No traditional game over	Living with HIV and missing ART are not a death sentence but one just a weakened immune system which can be managed	Failing to consume the artefact for five consecutive days results in the demon breaking free and resetting progress to the first stage.

### Game concept

**Fig 2:**
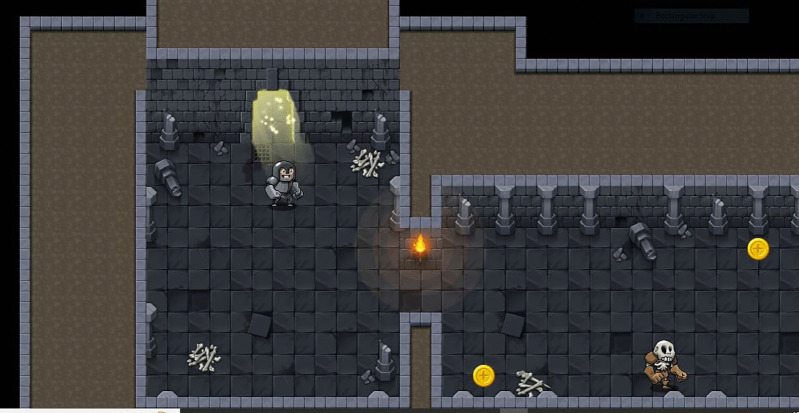
*The Conqueror* prototype.

*The Conqueror* (Fig 2) is an action or adventure game where the player controls a hero, “*The Conqueror*,” who is tasked with preventing the return of a demon that was sealed away years ago. Each day, the hero must battle minions, collect power-ups, and locate and pick a crucial artefact to keep the demon suppressed within three minutes of starting the game. Missing the artefact leads to increased difficulty, and failure to obtain it for five consecutive days results in the demon breaking free and resetting the player’s progress. The objectives of the games are to prevent the demon from breaking free by finding and consuming the artefact daily, battle minions, and collect power-ups to strengthen the hero, and progress through levels, each becoming brighter as the demon’s seals strengthen. The game features were guided by the SCT and IMB as indicated in Table 3 below.

**Table 3:** Integration of the theories of behaviour change principles into the game features.

Behaviour change theory principle	Application	Game feature	Significance or meaning
1. Knowledge	a. HIV knowledge	Prevention return of sealed demon	HIV can be conquered
	b. ART knowledge	Timed artefact collection	Importance of ART adherence (taking medication daily within the stipulated time)
2. Skill	Taking medication daily	Collecting artefact daily on time	Consistency in taking medication
3. Reinforcement	a. Positive	a. Pop-up messages	Support through encouragement
		b. Life bar	Suppressed viral load and increase in CD4 count
	a. Negative	a. Increase in difficulty	Unsuppressed viral load and decrease in CD4 count
		b. Resetting game to start	Compromised immunity, opportunistic infection
		c. Demon breaking lose	Death
4. Self-efficacy	Motivation and goal attainment	Killing minions while ensuring to collect artefact within 3 minutes of game start	Overcoming barriers and improved ART outcome
5. Expectation	Healthy, fulfilled adolescent	a. Progression to next stage	Acceptance (self and others)
		b. Suppressed demon	Suppressed viral load, decrease CD4 count

### Gameplay mechanics

a. Daily Cycle

The hero must enter the game at a specific time each day to battle minions and search for the artefact representing daily taking of medication. The artefact appears once a day and must be consumed to suppress the demon and clear the level. However, missing the artefact increases game difficulty and minion aggression. A day is compressed to ten minutes with ten lives.

b. Difficulty Progression

Within the first four days, missing the artefact each day increases the number and strength of minions while on the fifth day, the demon breaks free, punches the hero, and resets the game to the first stage.

c. Power-Ups

Various power-ups are scattered throughout the levels to aid the hero in battle. Power-ups include health boosts, strength enhancements, and speed increases.

d. Levels

Levels start with a darker theme, reflecting the weak state of the demon’s seals but as the hero progresses and successfully consumes the artefact daily, levels become brighter, symbolizing stronger seals on the demon.

e. Stages

### Stage 1: The Dark Forest

The first stage is a gloomy, forest filled with shadows and eerie sounds. In this stage, minions are relatively weak but numerous, providing a good challenge for new players. The stage takes place in a dark, misty environment with trees and underbrush with dim lighting and shadow effects to create a foreboding atmosphere. Basic health and strength boosts are hidden among the trees.

### Stage 2: The Cursed Village

The second stage is a dilapidated village with crumbling buildings and abandoned streets. Minions are stronger and more aggressive, reflecting the increased difficulty. The game takes place in ruined structures with overgrown vegetation and flickering lights. There is however slightly brighter environment compared to Stage 1, indicating progress. Further, enhanced strength and speed boosts are available in hidden spots.

### Stage 3: The Demon’s Lair

The third stage is the demon’s lair, a dark, cavernous environment with a foreboding presence. Minions are at their strongest, and the layout is more complex, challenging the player’s skills. The game takes place in a dark, rocky environment with lava flows and ominous lighting. However, this is the brightest stage compared to the previous ones, indicating near victory. Powerful boosts, including temporary invincibility and major health regeneration are available in this stage.

## Phase 4: Final functioning The Conqueror application

Once participants were satisfied, the game developer finalised the prototype to be ready for usability and acceptability testing by participants.

The final (Beta) version (–) of *The Conqueror* was developed and approved by participants for testing (https://snip.ly/shrjo9_android). Feedback from the last phase included participants’ eagerness to download and play the final version as quoted below:

**Fig 3:**
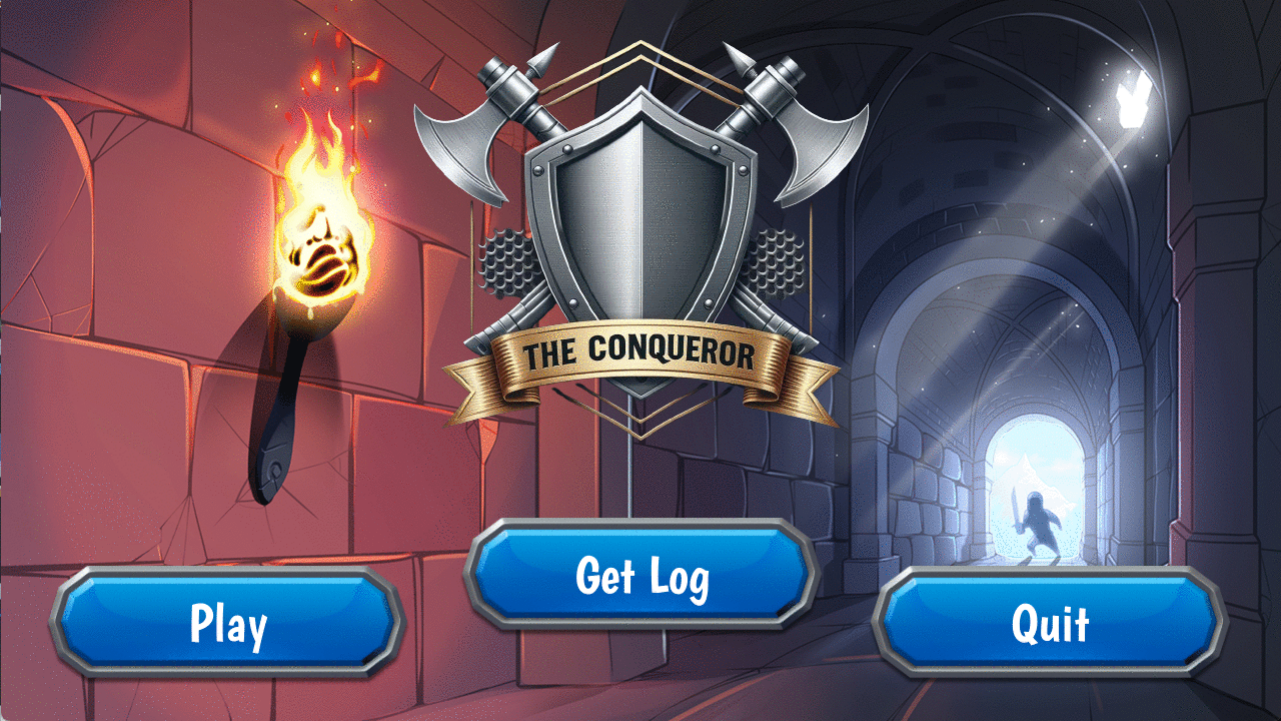
*The Conqueror* game homepage.

**Fig 4:**
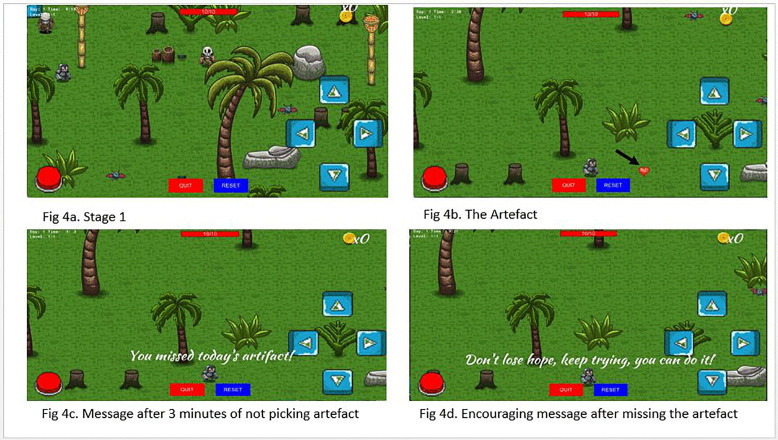
Stage 1 content of *The Conqueror* game.

**Fig 5:**
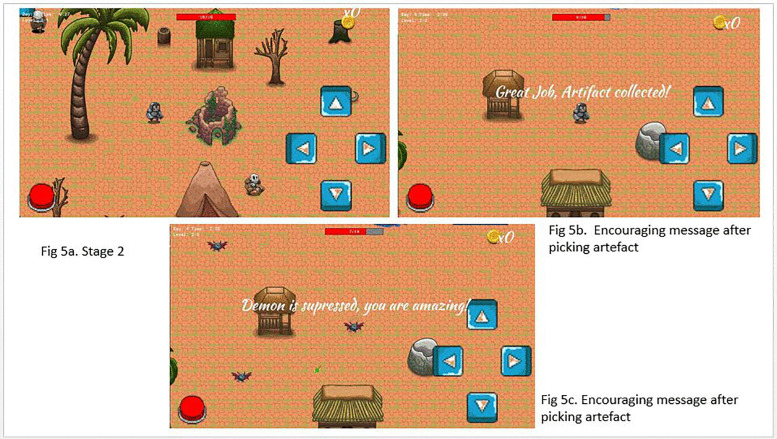
Stage 2 content and some encouraging messages.

**Fig 6:**
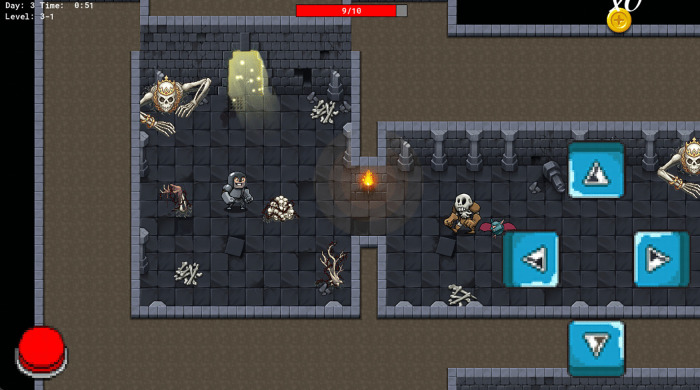
Stage 3 of *The Conqueror* game.

*“This is an amazing game and can’t wait to download and have it in my phone and most importantly, I am happy to be among the pioneers of such a ground-breaking project. I have one request though since by the time is rolled out, I won’t be a member of a Teen Club, can I be part of the team to implement in other health facilities”* Male ALHIV aged 19

## Phase 5: User acceptability testing

In this phase, the beta version of the game was presented to participants, who were given a week (6 days) to download, play, and critique it for usability and acceptability. This stage, referred to as external usability testing, aimed to assess whether participants could successfully download and navigate the game, understand its interface, interact with the prototype to explore its features, comprehend the educational concept, and evaluate the app’s engagement and relevance. Usability was defined as the user engagement, ease of use of the gaming application, while acceptability referred to the user’s perception, perceived appropriateness of the game, and their intent to recommend it to others. For usability and acceptability, data was collected quantitatively using a self-administered questionnaire and user metrics for both subjective and objective review. The questionnaire assessing game experience included a scoring form adapted from the client service questionnaire in which responses options were 1 = “Strongly agree”; 2 = “Agree”; 3 = "Neutral", 4=“Disagree”; and 5 = “Strongly disagree”). Secondly, user metrics were downloaded from administration panel for a comprehensive gaming review.

Quantitative data were entered into an Excel database. Frequency tabulations were done to assess for completeness and missing variables were corrected by cross checking with the data source. Data were imported to Stata 18 and descriptive analysis were conducted. Overall usability and acceptability of the mobile game was reported using the proportion of individuals’ score of each item in the form. Secondly, means of scores per item were calculated using total of scores divided by the number of participants and were reported with their standard deviations (SD). A mean score above three (3) which is the midpoint, was considered highly satisfactory while below the mid-point was considered non-satisfactory.

## Results

Out of the 35 respondents, 19 (54.3%) were female, and the majority (25/35; 71.4%) were aged between 15 and 19 years, as outlined in Table 4.

**Table 4:** Demographic characteristics of participants.

Variable		Frequency	Percentage
Sex	Female	19	54.3%
	Male	16	45.7%
Age	10 −14 years	10	28.6%
	15 −19 years	25	71.4%
Education	Primary	10	28.6%
	High school	23	65.7%
	Tertiary	1	2.9%
	Other	1	2.9%

### Overall game assessment

The overall game assessment received a rating above three, indicating a satisfactory evaluation by participants (Table 5). Most of the items were rated 4 (agree) by participants except for ease of download and the likelihood of recommending the game to others, which received a 5 (strongly agree) from the majority (Table 5). Ratings of 1 (strongly disagree) and 2 (disagree) were the least frequently given by participants across all items. Both age groups (10–14 years and 15–19 years) rated all aspects of the game as satisfactory (above 3), as shown in Table 6. However, younger adolescents provided slightly lower ratings for aspects such as ease of download, understanding, playability, ease of completion, ability to play without assistance, and the game’s balance of challenge and playability. In contrast, older adolescents expressed high satisfaction with the ease of downloading the game but assigned lower ratings to ease of play, completion, and ability to play without assistance (Table 7 and 8).

**Table 5:** Mean scores for all usability and acceptability variables.

Variable	StrD N (%)	D N (%)	N N (%)	A N (%)	SA N (%)	Mean score	SD
1. Easy to download	0 (0)	1 (2.9)	8 (22.9)	11 (31.4)	15 (42.9)	4.14/5	0.88
2. Easy to understand	0 (0)	0 (0)	9 (25.7)	17 (48.6)	9 (25.7)	4.00/5	0.73
3. Easy to play	1 (2.9)	1 (2.9)	7 (20)	19 (54.3)	7 (20)	3.86/5	0.88
4. Easy to complete	0 (0)	2 (5.71)	5 (14.3)	19 (54.3)	9 (25.7)	4.00/5	0.80
5. No assistance needed to play	0 (0)	2 (5.71)	8 (22.9)	18 (51.4)	7 (20)	3.86/5	0.81
6. Challenging but playable	0 (0)	0 (0)	6 (17.1)	16 (45.7)	13 (37.1)	4.20/5	0.72
7. Appealing	0 (0)	0 (0)	5 (14.3)	20 (57.1)	10 (28.6)	4.14/5	0.65
8. Comprehensive	0 (0)	0 (0)	6 (17.1)	17 (48.6)	12 (34.3)	4.17/5	0.71
9. Approve	0 (0)	1 (2.9)	4 (11.4)	17 (48.6)	13 (37.1)	4.20/5	0.76
10. Can recommend	0 (0)	1 (2.9)	5 (14.3)	13 (37.1)	16 (45.7)	4.26/5	0.83

StrD = strongly disagree, D = disagree, N = Neutral, A = agree, SA = strongly agree.

**Table 6:** Mean scores for all usability and acceptability variables stratified by age groups.

Items	10–14		15-19	
**mean score**	**SD**	**mean score**	**SD**
Easy to download	3.70/5	0.68	4.32/5	0.90
Easy to understand	3.90/5	0.74	4.04/5	0.74
Easy to play	3.70/5	0.95	3.92/5	0.86
Easy to complete	4.10/5	0.74	3.96/5	0.84
No assistance needed to play	3.60/5	0.84	3.96/5	0.79
Challenging but playable	3.80/5	0.79	4.36/5	0.34
Appealing	4.10/5	0.57	4.16/5	0.69
Comprehensive	4.00/5	0.67	4.24/5	0.72
Approve	4.00/5	0.67	4.28/5	0.79
Can recommend	4.50/5	0.53	4.16/5	0.10

**Table 7:** Game usability by participants.

Rating	Easy to download	Easy to understand	Easy to play	No assistance needed	Completed all stages with ease	Challenging but playable	Overall usability average %
StrD	0 (0)	0 (0)	1 (2.9)	0 (0)	0 (0)	0 (0)	0.48
D	1 (2.9)	0 (0)	1 (2.9)	2 (5.7)	2 (5.7)	0 (0)	2.9
N	8 (22.9)	9 (25.7)	7 (20)	8 (22.9)	5 (14.3)	6 (17.1)	20.4
A	11 (31.4)	17 (48.6)	19 (54.3)	18 (51.4)	19 (54.3)	16 (45.7)	47.6
SA	15 (42.9)	9 (25.7)	7 (20)	7 (20)	9 (25.7)	13 (37.1)	28.6
Total	35 (100)	35 (100)	35 (100)	35 (100)	35 (100)	35 (100)	100

**Table 8:** Game acceptability by participants.

Rating	Features appealing	Comprehensive	Approved	Can recommend	Overall acceptability average (%)
**Strongly disagree**	0 (0)	0 (0)	0 (0)	0 (0)	1.1
**Disagree**	0 (0)	0 (0)	1 (2.9)	1 (2.9)	2.9
**Neutral**	5 (14.3	6 (17.1)	4 (11.4)	5 (14.3)	13.1
**Agree**	20 (57.1)	17(48.6)	17 (48.6)	13 (37.1)	45.7
**Strongly agree**	10(28.6)	12(34.3)	13 (37.1)	16 (45.7)	37.1
**Total**	35 (100)	35 (100)	35 (100)	35 (100)	100

### Usability assessment

Usability was evaluated by assessing participants’ ability to download, understand, and play the game independently, navigate its features, and complete stages without assistance. On average, 86.2% of participants rated the game above average (agree and strongly agree) in terms of usability while 3.38% rated the below average (strongly disagree and disagree) as shown in Table 6. Notably, all variables for measuring usability were rated above average (50%) by majority of the participants with 82.8% rating the game challenging but playable, while only 80.0% of the participants allocated a “agree” and “strongly agree” to have been able to complete all stages with ease (Table 7).

Among participants aged 10–14 years, 65% agreed to strongly agree that the game was user-friendly while 31.7% were neutral on their rating (S1 Table). In contrast, 80.7% of participants aged 15–19 years believed the game was user-friendly (agree and strongly agree score) with 16% giving it a neutral rating (S1 Table). Generally, younger adolescents rated usability lower compared to older adolescents. For example, ease of download and ease of play were rated as “agree” or “strongly agree” by 60% of younger adolescents, compared to 80% of older adolescents. Similarly, 60% of younger adolescents, compared to 76% of older adolescents, agreed or strongly agreed that they could play without assistance.

### Acceptability Assessment

Acceptability was measured based on how engaging participants found the game features, their approval of gaming aids, the challenge level, and their willingness to recommend the game to others. Overall, game acceptance was high, with 82.8% of participants rating acceptability as 4 (agree) or 5 (strongly agree), while 4% rated it as unacceptable (disagree or strongly disagree) (Table 8). Younger adolescents demonstrated slightly higher acceptance levels, with 88% scoring acceptability as “agree” or “strongly agree” compared to 80.8% of older adolescents (S2 Table).

Younger adolescents found the game more appealing than older adolescents (90% and 60% respective), with 100% of younger participants indicating they would recommend the game to others, compared to 76% of older participants. Regarding comprehensiveness, 80% of younger adolescents provided a rating of “agree” or “strongly agree,” slightly lower than the 84% given by older adolescents (S2 Table).

## Discussions

*The Conqueror*, a gaming application designed to promote ART adherence among ALHIV in Eswatini, was developed using a user-centred, participatory approach. Grounded in behaviour change theories, including the IMB model and SCT, the game emphasizes the importance of knowledge, motivation, and self-efficacy in behaviour change, specifically ART adherence [30,33,36,53]. Behaviour change theories are important is identifying the underlying factors associated with behaviour change [30,36]. The game’s name, *The Conqueror*, was suggested by participants to represent their belief in their ability to conquer the challenges associated with ART adherence. This is to give a notation that HIV is not a death sentence as explained in a study conducted among people living with HIV [54]. Secondly, the name *The Conqueror* aligns with the goal of eliminating self-stigma associated with knowing one’s HIV status, a factor often linked to poor ART adherence among ALHIV [55]. Lastly, selecting a name that is not explicitly HIV-related is crucial for enhancing engagement while safeguarding against unintended status disclosure. This approach mirrors the strategy used in other games, such as WYZ, PeerNaija, AllyQuest which aim at addressing barriers to HIV care engagement among people living with HIV without explicitly referencing HIV [50,56–58]. Our study emphasises the importance of incorporating privacy measures in the design of mobile games aimed at promoting ART adherence. Ensuring privacy helps prevent unintended disclosure, which has been linked to poor adherence to ART [26].

Through its features, the game allows players to view themselves as heroes battling various barriers, with the primary goal of suppressing the virus load. Overcoming minor demons represents prevailing over ART adherence barriers. Similarly to the Epic Allies game, the storyline of *The Conqueror* centres on being a superhero, emphasizing the role of knowledge and empowerment in overcoming challenges related to ART adherence [59]. In Epic Allies, the superhero’s mission involves restoring the city of Medopolis to its former glory. In contrast, *The Conqueror* focuses on cultivating a positive attitude to overcome the obstacles faced by individuals living with HIV in maintaining ART adherence through killing minor demons [59]. Nevertheless, the superhero character underscores the importance of self-efficacy among people living with HIV in a quest to achieve ART adherence [60].

In *The Conqueror* game, players are encouraged to log in daily, with each in-game day compressed into ten minutes. Within the first three minutes, they must collect an artefact representing ART. Failure to do so on time results in increased strength of the main demon and heightened aggression from the minor demons, symbolizing the consequences of missing ART doses, including reduced immunity and eventual viral load suppression failure [61]. On the other hand, missing the artefact echoes missed ART doses, which lead to a weakened immune system, reduced CD4-count, and a non-suppressed viral load, ultimately leading to opportunistic infections and AIDS-related deaths [62,63]. Contrary to other games aimed at ART adherence, *The Conqueror* introduces a unique feature that emphasizes the critical timing of ART dosages [18]. This element serves as an educational tool, illustrating the consequences of missed doses by demonstrating their impact on the body. The concept was adapted from the design of Battle Viro, which focuses on defeating viruses to maintain strength. In *The Conqueror*, the emphasis shifts to the timing of medication, with missed doses depicted as leading to increased viral strength and aggression, ultimately highlighting the risk of ART resistance [64].

In the game, failure to collect the artefact results in a loss of life and a more challenging stage, but to encourage persistence, there is no traditional “game over.” Players are sent back to the beginning of the stage, reinforcing a message of support and perseverance, similar to findings of scoping review of interventions that emphasize the importance of psychosocial support in improving ART adherence among people living with HIV [65,66]. In addition to gameplay, *The Conqueror* includes encouraging messages, aligning with studies that have shown positive effects of messaging on ART adherence among adolescents [67–69]. These features were adapted from other games designed to enhance ART adherence, where information, motivation, and support play a critical role in driving behaviour change [50,59,70].

A review of gamification strategies identified seven key approaches: goal setting, enhancing the capacity to overcome challenges, providing performance feedback, offering reinforcement, enabling progress comparison, fostering social connectivity, and integrating fun and playfulness [18]. However, *The Conqueror* excluded the social connectivity aspect due to identified limitations, such as inconsistent network and internet access, which restrict constant connectivity in the feasibility of gamified interventions in Eswatini [26]. Social connectivity in the context of a mobile game refers to the game’s ability to foster social support, helping to reduce self-stigma, loneliness, isolation, and rejection, which are factors often linked to poor ART adherence among adolescents [71,72]. Within a mobile gaming framework, social connectedness can be achieved through multiplayer options that enable players to connect with other adolescents living with HIV [73]. This feature can help establish a secure social support system that promotes status acceptance while ensuring privacy and preventing unintended disclosure [26]. To address these constraints, the game was designed to support offline play. Notably, studies have reported 70% internet connectivity among young people, suggesting that incorporating both online and offline versions of the game could be particularly beneficial for older adolescents [13,15]. Additionally, the widespread use of and access to basic mobile phones among adolescents in Eswatini necessitated prioritizing the offline version [26]. Nevertheless, future iterations of the game will aim to include both online and offline versions to integrate the social connectivity strategy.

*The Conqueror* game was highly accepted by participants with over 80% participants rating it “agree” and “strongly agree” for both ease of use and perceived appropriateness and willingness to recommend to others. However, usability was lower among younger adolescents (65%) compared to older adolescents (80.7%). This disparity could be attributed to differing levels of prior exposure to games as explained in a study assessing feasibility and acceptability of video gaming to improve ART adherence [74]. Younger adolescents provided lower ratings for ease of downloading, understanding, and playing the game, whereas older adolescents rated aspects such as ease of play, completion, and independence (no need for assistance) slightly lower. The lower ratings could be attributed to varying levels of exposure and experience, as observed in studies conducted in Ghana, Nigeria, and Zimbabwe, where adolescents primarily used their phones for calling, texting, and taking photos [75 77]. The most commonly used applications were WhatsApp and Facebook, suggesting limited exposure to mobile gaming, which may explain the lower ratings in this study, particularly for aspects such as downloading, understanding, playing, and completing all stages without assistance [75,76]. Additionally, studies conducted in similar settings, such as Zambia and rural South Africa, have reported low levels of phone ownership among younger adolescents, with high rates of phone sharing within this age group [70,78]. Sharing phones with siblings, family members, or caregivers restricts adolescents’ exposure to phone features such as games, thereby limiting their familiarity with these functions [70,78]. In the South African study, younger adolescents were more likely to have access to basic phones rather than smartphones [78]. Since basic phones typically do not support gaming, this limited exposure may partly explain the lower usability rankings observed in this study. Conversely, in South Africa, older adolescents and young people were more likely to own smartphones and have access to private internet data, thereby expanding their opportunities for independent use of mobile features [78].

Nevertheless, the higher ratings in other aspects of game acceptance may be due to the game’s appealing features. Adolescents have been reported to prefer interactive elements such as points, leader boards, and playful mechanics, which likely contributed to the positive feedback on engagement and overall acceptance [75]. It is important to consider potential biases in these ratings such as social desirability bias, acquaintance and centre tendency biases associated with Likert scale measurements [79]. Social desirability suggests that participants may select higher ratings to present themselves in a favourable light. Additionally, acquaintance bias could lead participants to agree with presented ideas, potentially inflating scores. Lastly, central tendency bias, where participants gravitate toward neutral scores, may also affect the reliability of Likert scale measurements [79]. To address these limitations, incorporating other in-depth data collection tools, such as qualitative interviews or focus groups, is essential to better ascertain the usability of the game. This approach has been used successfully in usability testing for similar interventions, such as Ally Quest and Epic Allies [58,59].

Nevertheless, our study aligns with findings of a study conducted in Kenya which indicated high acceptability of smartphone games among adolescents [80]. The acceptability of an eHealth intervention is critical especially when conducted prior to implementation of the intervention to ensure effectiveness of the intervention [81]. However, while usability and acceptability were rated highly in this study, it is critical to consider the implementation and sustainability challenges associated with different adolescent age groups. These include access to mobile phones, type of device, usage patterns, and preferences for gaming applications. The findings emphasize the importance of a user-centred approach as essential in designing gamified interventions for ALHIV. They also highlight the feasibility of such interventions but point to potential limitations in effectiveness, particularly among younger adolescents who reported lower usability due to restricted phone access and prior exposure. Thus, critical consideration must be given to phone ownership, access, and usage patterns across age groups to ensure that gamified interventions meet the diverse needs and expectations of adolescents.

Moreover, the study highlighted the importance of actively involving adolescents in the design process to ensure their needs and preferences are addressed. For example, in this study the gaming app was intended to provide support through information and encouragement, while core gaming mechanisms included points, levels, badges, challenges and quests, as well as the use of a third-party agent such as an avatar representing the adolescent as noted in other studies [22,75]. Privacy and confidentiality also emerged as central concerns. Notably, while a previous study reported leaderboards being the most preferred feature, this feature was excluded in the present study due to concerns about unintended HIV status disclosure [82]. These findings reinforce the value of involving end-users in the design of gamified applications to enhance implementation, acceptability, and sustainability of such interventions.

### Strengths and limitations

This study has several notable strengths and limitations. A key strength is the use of a user-centred, participatory approach, emphasizing the importance of designing interventions with the end users rather than for them. This approach enhances both the acceptability and usability of the intervention. Additionally, the study employed multiple needs assessment methods, ensuring a comprehensive understanding of adolescents’ needs rather than limiting the scope to a narrowly defined population.

However, the study also has limitations. Firstly, the design of *The Conqueror* game involved only ALHIV conveniently sampled from one region of Eswatini. This may not fully represent the perceptions of ALHIV across the entire country, limiting the generalizability and transferability of the findings. Secondly, for the user assessment phase, only participants who owned mobile phones were included, potentially introducing selection bias, as adolescents without smartphones or prior phone usage experience were excluded. This may have overlooked the unique challenges and perspectives of these individuals. Additionally, the sample size was small, which may compromise the validity and generalizability of the results. Thirdly, the user acceptability assessment relied solely on a questionnaire (quantitative data), which measured overall acceptability but did not provide detailed insights into the functionality and user experience of the game. However, functionality was partially evaluated during real-time feedback sessions. Lastly, the game design did not incorporate input from healthcare providers, such as nurses and doctors, who could have offered valuable insights into medical accuracy and practical application.

Despite these limitations, the study successfully captured the perspectives of end users. In future phases, healthcare providers will be engaged to enrich the intervention and ensure its alignment with existing HIV management programs for ALHIV, thereby enhancing the potential for feasibility and integration. Additionally, a randomized trial will be conducted to pilot the mobile game, assessing both its feasibility and effectiveness.

## Conclusion

A user-led, participatory approach is essential when designing interventions for adolescents, as their needs and perspectives differ significantly from those of other population subgroups. The widespread availability of mobile phones offers a valuable opportunity to co-design gamified interventions that are both engaging and acceptable to ALHIV. In this study, we successfully co-designed a gamified intervention aimed at improving ART adherence among ALHIV.

However, further research is needed to evaluate the feasibility and effectiveness of this gaming application in enhancing ART adherence among adolescents in Eswatini, with the potential for scaling up to reach broader populations. The feasibility of the intervention relies on the involvement of the Ministry of Health and other key stakeholders to explore the integration of the mobile application within Teen Clubs and other adolescent HIV management programs.

Additionally, the feasibility assessment must address critical factors such as phone ownership, device compatibility, and other elements inherent to gaming interventions, including internet access. These considerations will help refine the final gaming application mechanics, ensuring its practicality and effectiveness in promoting ART adherence.

## Supporting information

S1 Fig Storybook by participants aged 10–14 years. (TIF)

S2 Fig Storybook by participants aged 15–19 years. (TIF)

S1 Table Game usability stratified by participants’ age group. (DOCX)

S2 Table Game acceptability stratified by participants” age group. (DOCX)

S1 File Inclusivity in global research questionnaire. (DOCX)

## Abbrevia`tions

HIV: Human immunodeficiency virus
AIDS: Acquired immunodeficiency syndrome
ALHIV: adolescents living with HIV
ART: antiretroviral therapy
CD4: cluster of differentiation 4
SSA: sub-Saharan Africa
VLS: Viral load suppression
VL: Viral load
IMB: Information motivation behavioural skill
SCT: Social cognitive theory
FGDs: Focused group discussions
