# Supplementary material for: A mobile game to promote ART adherence among adolescents living with HIV in Eswatini: Development and prototype testing of “The Conqueror”
Source: PLoS One. 2026 Feb 5;21(2):e0321907. doi: 10.1371/journal.pone.0321907 (PMC12875499; doi:10.1371/journal.pone.0321907)
Supplement: S1 Table — (DOCX) [file pone.0321907.s003.docx]

| **Rating** | **Easy to download** | | **Easy to understand** | | **Easy to play** | | **No assistance needed** | | **Completed all stages with ease** | | **Challenging but playable** | | **Overall usability average (%)** | |
| --- | --- | --- | --- | --- | --- | --- | --- | --- | --- | --- | --- | --- | --- | --- |
|  | **10-14 N (%)** | **15-19 N (%)** | **10-14 N (%)** | **15-19 N (%)** | **10-14 N (%)** | **15-19 N (%)** | **10-14 N (%)** | **15-19 N (%)** | **10-14 N (%)** | **15-19**  **N (%)** | **10-14 N (%)** | **15-19 N (%)** | **10-14 (%)** | **15-19 N (%)** |
| **StrD** | 0 (0) | 0 (0) | 0 (0) | 0 (0) | 0 (0) | 1 (4) | 0 (0) | 0 (0) | 0 (0) | 0 (0) | 0 (0) | 0 (0) | 0 | 0.7 |
| **D** | 0 (0) | 1 (4) | 0 (0) | 0 (0) | 1 (10) | 0 (0) | 1 (10) | 1 (4) | 0 (0) | 2 (8) | 0 (0) | 0 (0) | 3.3 | 2.7 |
| **N** | 4 (40) | 4 (16) | 3 (30) | 6 (24) | 3 (30) | 4 (16) | 3 (30) | 5 (20) | 2 (20) | 3 (12) | 4 (40) | 2 (8) | 31.7 | 16 |
| **A** | 5 (50) | 6 (24) | 5 (50) | 12 (48) | 4 (40) | 15 (60) | 5 (50) | 13 (52) | 5 (50) | 14 (56) | 4 (40) | 12 (48) | 46.7 | 48 |
| **SA** | 1 (10) | 14 (56) | 2 (20) | 7 (28) | 2 (20) | 5 (20) | 1 (10) | 6 (24) | 3 (30) | 6 (24) | 2 (20) | 11 (44) | 18.3 | 32.7 |
| **Total** | 10 (100) | 25 (100) | 10 (100) | 25 (100) | 10 (100) | 25 (100) | 10 (100) | 25 (100) | 10 (100) | 25 (100) | 100 (100) | 25 (100) | 100 | 100 |

S1 Table. Usability stratified by age group
