# Supplementary material for: A mobile game to promote ART adherence among adolescents living with HIV in Eswatini: Development and prototype testing of “The Conqueror”
Source: PLoS One. 2026 Feb 5;21(2):e0321907. doi: 10.1371/journal.pone.0321907 (PMC12875499; doi:10.1371/journal.pone.0321907)
Supplement: S2 Table — (DOCX) [file pone.0321907.s004.docx]

S2 Table. Game acceptability stratified by age group

| **Rating** | **Features appealing** | | **Comprehensive** | | **Approved** | | **Can recommend** | | **Overall acceptability average (%)** | |
| --- | --- | --- | --- | --- | --- | --- | --- | --- | --- | --- |
|  | **10-14**  **N (%)** | **15-19**  **N (%)** | **10-14**  **N (%)** | **15-19**  **N (%)** | **10-14**  **N (%)** | **15-19**  **N (%)** | **10-14**  **N (%)** | **15-19**  **N (%)** | **10-14**  **(%)** | **15-19**  **(%)** |
| **Strongly disagree** | 0 (0) | 0 (0) | 0 (0) | 0 (0) | 0 (0) | 0 (0) | 0 (0) | 0(0) | 0 | 1.6 |
| **Disagree** | 0 (0) | 0 (0) | 0 (0) | 0 (0) | 0 (0) | 1 (4) | 0 (0) | 1 (4) | 0 | 4 |
| **Neutral** | 1 (10) | 4 (16) | 2 (20) | 4 (16) | 2 (20) | 2 (8) | 0 (0) | 5 (20) | 12 | 13.6 |
| **Agree** | 7 (70) | 13 (52) | 6 (60) | 11(44) | 6 (60) | 11 (44) | 5 (50) | 8 (32) | 58 | 40.8 |
| **Strongly agree** | 2 (20) | 8 (32) | 2 (20) | 10 (40) | 2 (20) | 11 (44) | 5 (50) | 11 (44) | 30 | 40 |
| **Total** | 10 (100) | 25 (100) | 10 (100) | 25 (100) | 10 (100) | 25 (100) | 10 (100) | 25 (100) | 100 | 100 |
